# Oleacein Intestinal Permeation and Metabolism in Rats Using an In Situ Perfusion Technique

**DOI:** 10.3390/pharmaceutics13050719

**Published:** 2021-05-14

**Authors:** Anallely López-Yerena, Maria Pérez, Anna Vallverdú-Queralt, Eleftherios Miliarakis, Rosa M. Lamuela-Raventós, Elvira Escribano-Ferrer

**Affiliations:** 1 Department of Nutrition, Food Science and Gastronomy XaRTA, Faculty of Pharmacy and Food Sciences, Institute of Nutrition and Food Safety (INSA-UB), University of Barcelona, 08028 Barcelona, Spain; naye.yerena@gmail.com (A.L.-Y.); mariaperez@ub.edu (M.P.); avallverdu@ub.edu (A.V.-Q.); lamuela@ub.edu (R.M.L.-R.); 2 Laboratory of Organic Chemistry, Faculty of Pharmacy and Food Sciences, University of Barcelona, 08028 Barcelona, Spain; 3 CIBER Physiopathology of Obesity and Nutrition (CIBEROBN), Institute of Health Carlos III, 28029 Madrid, Spain; 4 Department of Chemistry, Voutes Campus, University of Crete, 70013 Heraklion, Greece; leytmil@gmail.com; 5 Biopharmaceutics and Pharmacokinetics Unit, Department of Pharmacy and Pharmaceutical Technology and Physical Chemistry, Faculty of Pharmacy and Food Sciences, Institute of Nanoscience and Nanotechnology (IN2UB), University of Barcelona, 08028 Barcelona, Spain; 6 Pharmaceutical Nanotechnology Group I+D+I Associated Unit to CSIC, University of Barcelona, 08028 Barcelona, Spain

**Keywords:** bioavailability, extra virgin olive oil, secoiridoids, metabolism, phenolic compounds, intestinal permeability

## Abstract

Oleacein (OLEA) is one of the most important phenolic compounds in extra virgin olive oil in terms of concentration and health-promoting properties, yet there are insufficient data on its absorption and metabolism. Several non-human models have been developed to assess the intestinal permeability of drugs, among them, single-pass intestinal perfusion (SPIP), which is commonly used to investigate the trans-membrane transport of drugs in situ. In this study, the SPIP model and simultaneous luminal blood sampling were used to study the absorption and metabolism of OLEA in rats. Samples of intestinal fluid and mesenteric blood were taken at different times and the ileum segment was excised at the end of the experiment for analysis by LC–ESI–LTQ–Orbitrap–MS. OLEA was mostly metabolized by phase I reactions, undergoing hydrolysis and oxidation, and metabolite levels were much higher in the plasma than in the lumen. The large number of metabolites identified and their relatively high abundance indicates an important intestinal first-pass effect during absorption. According to the results, OLEA is well absorbed in the intestine, with an intestinal permeability similar to that of the highly permeable model compound naproxen. No significant differences were found in the percentage of absorbed OLEA and naproxen (48.98 ± 12.27% and 43.96 ± 7.58%, respectively).

## 1. Introduction

The small intestine is the main site for drug absorption after oral administration [1,2], and the intestinal epithelial membrane is the principle physiological barrier that chemicals must cross to enter the bloodstream and become bioavailable [3]. Many in silico, in vitro, in situ, and in vivo models have been developed to investigate the transport mechanisms, intestinal permeability, and plasma pharmacokinetic profile of chemicals [4]. The in vivo models are the most clinically relevant because they include all the physiological factors that can affect absorption and bioavailability [1,4], but they are less useful for mechanistic studies. The in situ models, such as single-pass intestinal perfusion (SPIP), are commonly used to investigate trans-membrane chemical transport. SPIP allows for intestinal effective permeability to be determined on the basis of the disappearance of the compound from the intestinal segment or its appearance in plasma after a venous sampling procedure [5,6,7,8,9], with the latter being especially suitable for poorly permeating substances [8]. Moreover, SPIP is the most similar alternative to an in vivo model in that it includes a mucus layer, blood irrigation, and innervation [4,6,7].

Intestinal permeability depends on the physicochemical properties and molecular structure and size of the drug or other xenobiotic molecules [2]. Lipophilicity, solubility, and the acid–base character can also affect the rate and extent of absorption, distribution, and transport through biological membranes [3]. In addition, the absorption rate is affected by the expression of transporter proteins and enzymes [1,3,10,11] that are highly region-dependent [2] and other biological factors [2,12].

The benefits of dietary phenolics have been extensively reported [13,14], especially as a protection from cardiovascular diseases. In extra virgin olive oil (EVOO), secoiridoids such as oleacein (OLEA) represent by far the most abundant group of phenolic compounds (>90%) [15] and are thought to be responsible for the healthy properties of the oil, although mediated by other components. In the review by Naruszewicz et al. (2015), OLEA is described as a substance with high pharmacological potential [16]. As well as various antioxidant and anti-inflammatory properties [17,18], OLEA exhibits anti-proliferative and anti-metastatic effects in the SH-SY5Y human neuroblastoma cell line [19].

Numerous studies have explored the bioavailability of phenolic compounds after the ingestion of EVOO or a phenolic extract, both in humans [20,21,22,23,24,25] and rats [26,27,28,29]. However, among the secoiridoids, most attention has been focused on oleuropein and hydroxytyrosol and its derivatives [30,31], and little information is available on OLEA. Thus, further animal and human studies using purified OLEA are needed to clarify whether the biological effects attributed to it are due to OLEA itself or its metabolites. In addition, as explained above, in situ studies are required to obtain detailed information on the intestinal permeability of OLEA and to verify the potential sites of its metabolization after oral administration.

To the best of our knowledge, no studies have been previously performed on pure OLEA using SPIP in rats. With this model, OLEA and its metabolites were simultaneously monitored in the intestinal lumen and mesenteric blood plasma, with the aim of shedding light on its intestinal permeability and metabolism. The resulting information will be useful for a better understanding of the biological effects attributed to OLEA. The highly permeable naproxen was included in the study as a reference drug.

## 2. Materials and Methods

### 2.1. Reagents and Materials

OLEA (≥90% purity) was purchased from Toronto Research Chemicals (North York, ON, Canada). Naproxen and phenol red were purchased from Sigma-Aldrich (Madrid, Spain). Heparin sodium salt from porcine intestinal mucosa, Hanks’ balanced salt solution (HBBS), and HEPES 1M solution were also obtained from Sigma-Aldrich (Madrid, Spain). Pentobarbital sodium 200 mg/mL (Dolethal) was supplied by Vetoquinol (Madrid, Spain) and isoflurane by Laboratorios Esteve (Barcelona, Spain). The solvents acetonitrile and methanol, and the chemical formic acid were acquired from PanReac AppliChem (Panreac Quimica SLU, Barcelona, Spain). Finally, a Milli-Q purification system was used to obtain ultrapure water (Millipore, Bedford, MA, USA).

### 2.2. Work Solutions

**Figure 1:**
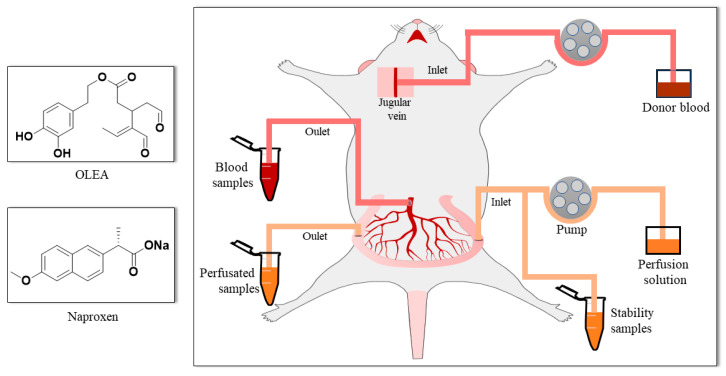
Chemical structure of the perfused compounds (OLEA and naproxen) and surgical procedure.

The transport medium (TM; pH 7, 9.7 g/L HBSS buffered with HEPES 10 mM) was used to infuse the compounds via the intestine. The secoiridoid OLEA (Figure 1) was assayed at 0.15 mg/mL (468.3 µM), a concentration chosen after taking into account both the OLEA concentration in EVOO (around 300 mg/kg) and the daily ingestion of EVOO recommended by the European Food Safety Authority (EFSA) (at least 5 mg of hydroxytyrosol and its derivatives per 20 g of olive oil) [32]. In accordance with its high dose strength, naproxen was assayed at 2.2 mg/mL in 250 mL of TM, as recommended [33]. Red phenol was assayed at 0.1 mg/mL. The stability of these test compounds, sampled at different times, was monitored in the perfusion solution at 37 °C for 60 min.

### 2.3. Animals

The Animal Experimentation Ethics Committee of the University of Barcelona, Spain (trial no. CEEA 124/16), and Generalitat de Catalunya (no. 6435, 27 June 2019) approved the study protocol. For each compound, four male Sprague-Dawley rats (Envigo RMS Spain SL, Sant Feliu de Codines, Barcelona, Spain) were used per intestinal perfusion experiment and four as blood donors (body weight 285 ± 6 g and 300–400 g, respectively). The animals arrived at the animal facility 10 days before the experiment and were kept with water and food ad libitum, with a 12 h light and dark cycle, at 21 °C and humidity-controlled (55 ± 10%).

### 2.4. Single-Pass Intestinal Perfusion Studies

The SPIP studies were performed in anesthetized rats according to the method described by López-Yerena and coworkers [6]; the donor blood and surgical procedure (Figure 1) were as described in that study. Regarding the procedure of donor blood, the rats were anesthetized by isoflurane inhalation (2.5%), and the whole blood was collected via cardiac puncture. The blood was diluted with heparin (50 u/mL TM) to 80% blood and kept in a 20 mL syringe for the in situ SPIP experiment. In the case of the rats that underwent surgery, the rats received intraperitoneal administration of pentobarbital sodium (Dolethal) at a dose of 60 mg/kg, and a maintenance dose of isoflurane (1.5%) was given 50 min after the induction dose. Body temperature was maintained at 37 ± 0.5 °C throughout the experiment by means of a homeothermic blanket. An ileal segment of approximately 7–10 cm was cannulated. The intestinal perfusion was started by delivering the perfusion solution containing OLEA or naproxen (in separate experiments) and phenol red at a flow rate of 0.2 mL/min to the cannulated intestinal segment, and the blood was supplied though the jugular vein at a rate of 0.3 mL/min. The phenol red included in the perfusion solution was used as non-absorbable marker for measuring water transport. To avoid the oxidation of OLEA, we carried out the SPIP experiments in a laboratory room with infrared light. Samples of both the intestinal lumen and mesenteric blood were collected simultaneously at 5 min intervals for 60 min. The outflow perfusate samples were collected in 2 mL amber vials and the blood was collected in pre-weighed lithium-heparinized tubes (BD Vacutainer). Next, the perfusate samples were centrifuged (7516× *g* for 10 min at 4 °C), and the tubes with blood samples were weighed and centrifuged (3000× *g* for 10 min at 4 °C). The supernatants were collected and immediately stored at −80 °C awaiting analysis by LC–ESI–LTQ–Orbitrap–MS.

### 2.5. Biological Sample Treatment

Plasma: The extraction of OLEA and its metabolites was carried out with protein precipitation. Initially, the samples were thawed and centrifuged (11,000× *g*, 10 min at 4 °C). To precipitate proteins, we blended 100 µL of plasma with cold acetonitrile containing 2% of formic acid (1:5 *v*/*v*). Samples were vortex-mixed for 1 min and kept at −20 °C for 20 min. The samples were then centrifuged (11,000× *g*, 4 °C, 10 min), and finally 100 µL of the supernatant was transferred to vials for analysis.

With regard to naproxen, the plasma samples were deproteinized following the methodology proposed by Elsinghorst and colleagues [34], with some modifications. Rat plasma samples (100 µL) were deproteinized by the addition of 200 µL of acetonitrile. After thorough vortex-mixing, the samples were centrifuged (2733× *g*, 4 °C, 10 min), and the supernatant was mixed with 0.02 M ammonium acetate buffer (pH 4.0) (1:1 *v*/*v*). Again, they were vortex-mixed and after centrifugation, and 100 µL of the supernatant was transferred to vials for analysis by LC–MS/MS.

Stability study and lumen samples: The stability and perfusate OLEA samples were defrosted and centrifuged (11,000× *g*, 4 °C, 10 min), and 100 µL of the upper phase was transferred to vials for analysis. The equivalent naproxen samples were thawed and centrifuged (11,000× *g*, 4 °C, 10 min), and the upper phase was diluted with TM in a ratio of 5:95 (stability samples) and 1:9 (*v*/*v*) (perfusate samples) and transferred to vials.

Ileum tissue: The intestinal segment perfunded with OLEA in the SPIP experiments was also analyzed to quantify the OLEA and its metabolites retained in the tissue. First, the intestinal segment was rinsed by several perfusions with TM and then cut into small sections and homogenized after the addition of water/acetonitrile (1:1 (*v*/*v*) with 0.1% ascorbic acid) with a small tissue disruptor (T10 basic ULTRA-TURRAX^®^, IKA laboratory technology, Staufen, Germany). The samples were sonicated in an ice bath (5 min), shaken for 1 min, and centrifuged (11,000× *g*, 4 °C, 10 min). To precipitate proteins, we blended 100 µL of the upper layer with cold acetonitrile containing 2% of formic acid (1:3 *v*/*v*) [35]. Samples were vortex-mixed for 1 min, kept at −20 °C for 20 min, and centrifuged again (11,000× *g*, 4 °C, 10 min) before analyzing the supernatant.

### 2.6. Analytical Technique

#### 2.6.1. OLEA Analysis

The quantification of OLEA and red phenol in the samples and the profiling and structural characterization of OLEA metabolites was carried out using LC–ESI–LTQ–Orbitrap–MS. LC separation was performed using an Accela chromatograph (Thermo Scientific, Hemel Hempstead, UK) equipped with a quaternary pump, a photodiode array detector, and a thermostated autosampler. A 5-μL sample aliquot was injected onto an Acquity^TM^ UPLC^®^ BEH C_18_ Column (2.1 × 100 mm, i.d., 1.7 µm particle size) coupled to an Acquity^TM^ UPLC^®^ BEH C_18_ Pre-Column (2.1 × 5 mm, i.d., 1.7 µm particle size) (Waters Corporation^®^, Wexford, Ireland) with the column temperature set at 50 °C. Eluent A was 0.05% (*v*/*v*) formic acid in water, and eluent B was 0.05% formic acid in methanol. The total run time was 11 min. The elution gradient (0.6 mL/min) started at 0% B and was increased via a linear gradient to 53.6% B after 6 min. The gradient was then increased for 2 min to 100% B and held for 1 min before returning to 0% B for 1.9 min to re-equilibrate the column.

The mass spectrometer used for the analysis was an LTQ Orbitrap Velos (Thermo Scientific, Hemel Hempstead, UK) equipped with an electrospray (ESI) source. The ESI source was operated in negative mode [M–H] with the following conditions: source voltage, 4 kV; capillary temperature, 275 °C (FT Automatic gain control (AGC) target 5·105 for MS mode and 5·104 for MS^n^ mode); sheath gas (ultra-pure nitrogen, >99.9%); flow rate 20; auxiliary gas flow rate 10; and sweep gas flow rate 2. In the case of the last 3 parameters, the arbitrary units were initially used, Fourier transform mass spectrometry (FTMS) mode was then used to analyze at a resolving power of 30,000 at *m*/*z* 600, and the data-dependent MS/MS events were acquired at a resolving power of 15,000 at *m*/*z* 600. The most intense ions detected in FTMS mode triggered data-dependent scanning. Ions that were not intense enough for a data-dependent scan were analyzed in MS^n^ mode with the same orbitrap resolution (15,000 at *m*/*z* 600). Precursors were fragmented by collision-induced dissociation (CID) using a C-trap with normalized collision energy (35 V) and an activation time of 10 ms. The mass range in FTMS mode was from 100 to 600 (*m*/*z*). The system was controlled by Xcalibur 3.0 software (ThermoFisher Scientific, Hemel Hempstead, UK).

Accurate masses and the isotopic pattern (through the Formula Finder feature in Xcalibur 3.0 software (ThermoFisher Scientific, Hemel Hempstead, UK) were used to select the elemental composition of each OLEA derivative. In addition, metabolites were confirmed by comparison with those reported in the literature [21,22] and with a similar compound [6,36,37]. MS^n^ measurements were performed to obtain information about fragment ions generated in the linear ion trap within the same analysis.

The OLEA and phenol red calibration curves were prepared in TM (10–150 µg/mL). The OLEA calibration curves were also prepared in rat plasma (0.1–3 µg/mL) and ileum tissue (0.1–3 µg/mL). All calibration curves had an *R^2^* > 0.97. In the absence of a reference standard, OLEA derivatives were evaluated by considering the ratio between peak area metabolite and parent compound dosed (OLEA) [6].

#### 2.6.2. Naproxen Analysis

All luminal, plasma, and stability samples were analyzed by ultra-high-performance liquid chromatography/ESI tandem mass spectrometry (UHPLC–ESI–MS/MS) following the procedure proposed by Elsinghorst et al. with some modifications [34]. The liquid chromatography system consisted of an Acquity^TM^ UPLC (Waters; Milford, MA, USA). Chromatographic separations were performed on an XBridge^TM^ C_18_ (4.6 × 50 mm, 5µm particle size) column (Waters Corporation^®^, Wexford, Ireland). The mobile phase consisted of 0.02 M ammonium acetate buffer (pH 4.0) and acetonitrile (30/70, *v*/*v*) and was delivered at a flow rate of 1.0 mL/min (column temperature at 30 °C). A total of 10 µL of sample was injected.

The detection and quantification of naproxen and red phenol were performed using an AB SCIEX API 3000^TM^ triple quadrupole mass spectrometer with a turbo ion spray source. Ionization was performed by ESI in the negative mode [M–H] in the multiple monitoring mode (MRM). Arbitrary units were used for the nebulizer (10), curtain (12), and drying gas (450 °C) using N_2_; the capillary voltage was −3500 V. To detect naproxen and red phenol with the highest signal, we optimized the collision energy and the declustering, focusing, and entrance potential by direct infusion. The system was operated by Analyst version 1.4.2 software supplied by ABSciex (ABSciex, Framingham, MA, USA).

The calibration curves with naproxen and phenol red were prepared in TM (10–150 µg/mL) and in rat plasma (0.5–20 µg/mL). The samples were adequately diluted to be interpolated in the calibration curves. All calibration curves had an *R*^2^ > 0.98.

### 2.7. Data Analysis

The equations used to determine the effective permeability coefficient, the correction of outlet concentrations, and the apparent permeability coefficient through the ileum are as follows:

(1) $$P_{\mathrm{eff}}=\frac{-{Ø}_{in}}{2*\;\pi *R*L}*Ln\;\frac{C_{out.cor}}{C_{in}}$$

(2) $$C_{out.cor}=C_{out}*\frac{CPR_{in}}{CPR_{out}}$$

(3) $$P_{\mathrm{app}}=\frac{dQ}{dt}*\frac{1}{A*C_{0}}$$

where ${Ø}_{in}$ is the perfusion solution flow (0.2 mL/min), *C_in_* and *C_out.cor_* are the respective inlet and corrected outlet steady-state concentrations of the tested product, *R* is the radius of the intestinal segment (set to 0.2 cm), and *L* is the length of intestinal segment determined after completion of the perfusion experiment.

The outlet concentrations were corrected for water transport by measuring the phenol red (*PR*) marker according to Equation (2), where *C_out_* is the concentration of OLEA or naproxen in the perfusate at the specified time interval, and *CPR_in_* and *CPR_out_* are the phenol red concentrations in the inlet and outlet solutions at the specific time intervals, respectively.

The *P_app_* was calculated using Equation (3), where *Q* is the cumulative number of tested compounds (OLEA or naproxen) appearing in the mesenteric blood as a function of time *t* in steady state conditions, *A* is the surface area of the exposed intestinal segment, and *C*_0_ is the tested compounds initial concentration in the perfusate.

All in situ perfusion experiments were replicated in four rats. Data are presented as the arithmetic mean ± the standard deviation (SD). Statistical analysis was performed using Statgraphics Centurion XVI software (Statpoint Technologies Inc., Warrenton, VA, USA). The concentrations estimated at a range of times (stability samples) and the concentration of metabolites and OLEA at different times were compared using a parametric statistical assay (ANOVA test). Statistical differences in the concentration of metabolites between plasma and perfusion samples were analyzed using were evaluated using an ANOVA test, followed by the LSD post hoc test. The *P*_eff_ and *P*_app_ of OLEA and naproxen were compared using a Mann–Whitney *U* test. Differences were considered significant at *p* < 0.05.

## 3. Results and Discussion

### 3.1. Stability of OLEA

The stability of OLEA and the reference standard naproxen was evaluated prior to carrying out the intestinal permeability study, and both remained stable in the perfusion solution at 37 °C (*p* > 0.05) for the length of the experiments, i.e., over 60 min (Figure S1). The absence of degradation products such as M2 and elenolic acid was also verified, although traces of a hydrated form of OLEA (M5) were detected, probably due to interaction of the aldehyde groups with the aqueous TM during the analysis.

### 3.2. Qualitative and Quantitative Characterization of OLEA and Its Metabolites

The lumen, plasma, and ileum samples were analyzed by HPLC–ESI–LTQ–Orbitrap–MS to identify OLEA and its metabolites. The FTMS scan and MS^n^ experiments allowed for the identification of OLEA (M1), four phase I metabolites (M2, M3, M4, and M5), and six phase II metabolites (M6, M7, M8, M9, M10, and M11). These metabolites and their precursor ions (measured), tentative formula, mass error, retention times, and major fragments are presented in Table 1. An example of a chromatogram for each metabolite and the parent compound is shown in the supporting information (Figure S2), and the structure for the detected fragments is also proposed in Figure S3.

**Figure 2:**
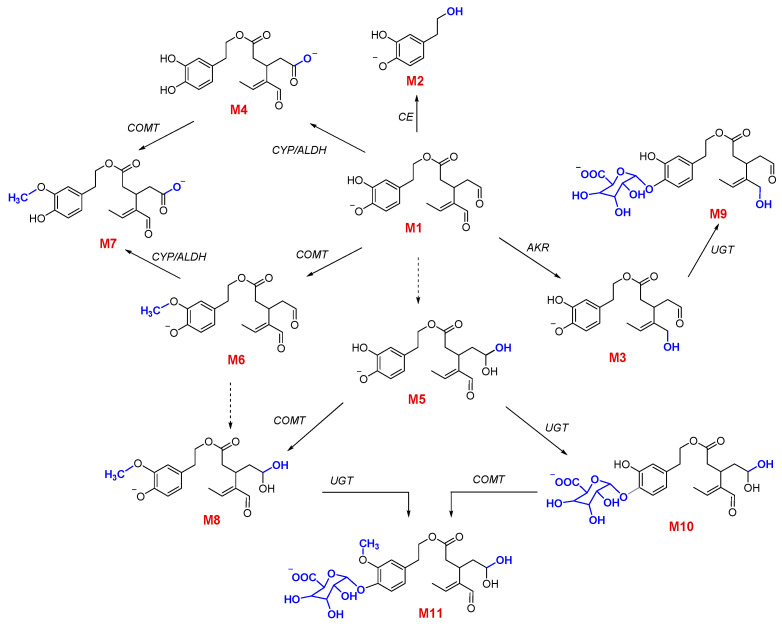
Proposed metabolic pathway of OLEA (M1) with phase I and phase II reactions. The chemical structures of M1 to M11 were identified in the plasma, lumen, and/or ileum samples after the SPIP study. CE: carboxylesterases; AKR: aldo-keto reductases; CYP3A: subfamily of cytochrome P450 enzymes; UGTs: glucuronosyltransferases; COMT: catechol-O-methyltransferase; ALDH: aldehyde dehydrogenase.

OLEA metabolites have been studied previously, but their structures have not been reported [21,22]. As mentioned above, on the basis of the fragmentation pattern of each detected metabolite and in comparison with related phenolic compounds [6,36], we proposed tentative structures for the OLEA derivatives (Figure 2).

### 3.3. Phase I Metabolism

**Figure 3:**
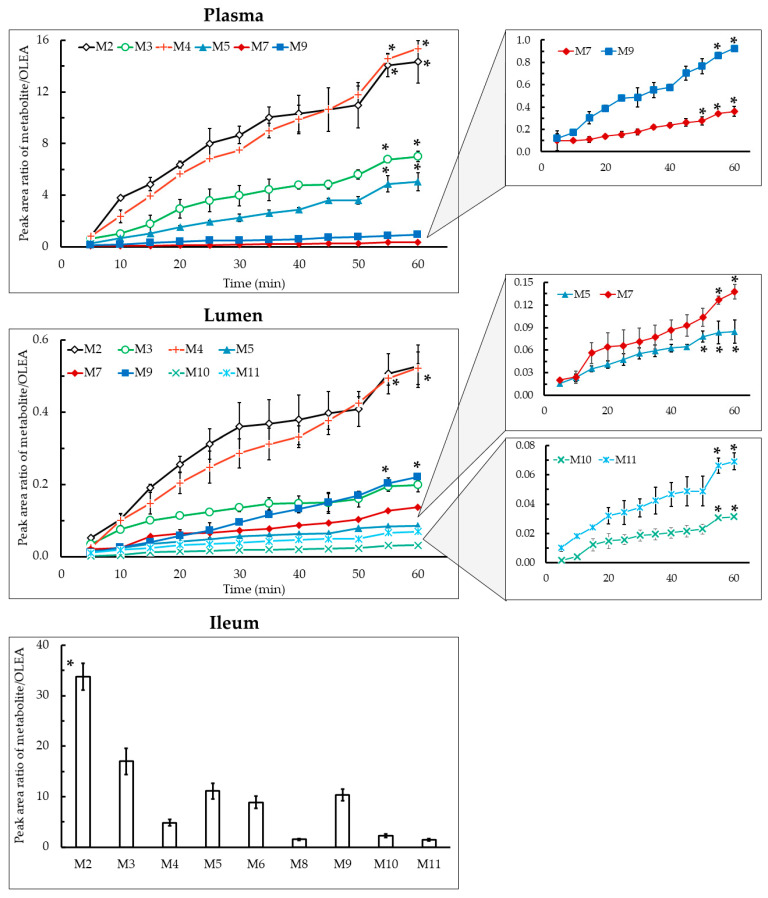
Peak area ratio of metabolite/OLEA as a function of time in plasma, lumen, and ileum tissue samples. Results are expressed as the mean ± standard deviation. * *p* < 0.05, one-way ANOVA.

OLEA metabolites arising from phase I reactions both in lumen and plasma samples are shown in Figure 3. For the first time, the metabolic profile of OLEA in the ileum of rats after an SPIP assay has been evaluated. It is worth noting that the metabolite levels were much higher in plasma than the lumen, e.g., levels of M2 and M4 were 27- and 29-fold higher at 60 min, respectively. In the ileum tissue samples, the main metabolite was M2 (*p* < 0.05). The main phase I metabolites detected in plasma and lumen samples were M2 and M4, and its concentration was favored in time reaching the higher concentration at 55 and 60 min, respectively (*p* < 0.05). The large number of metabolites identified and their relatively high abundance (peak area metabolite/parent ratio up to 15 in plasma) indicates an important intestinal first-pass effect during the absorption of OLEA and that the metabolites formed are mainly transferred to the systemic circulation.

Hydrolysis is known to be a common process in phase I drug metabolism. Carboxylesterases (CEs) catalyze the hydrolysis of esters, thioesters, amides, and carbamates, with carboxylic acids and alcohols as the hydrolysis products [38,39]. Among the enzymes of this family, CES2 is present in the small intestine in both humans and rats [40]. The hydrolysis of OLEA can lead to the formation of hydroxytyrosol (M2) and elenolic acid [27]. In our study, M2 was found in lumen, plasma, and ileum tissue, but elenolic acid was not detected in any sample. Similar results were obtained in the study of Pinto et al. (2011), where the M2 metabolite was detected in Caco-2 cells and in the in vitro study of isolated intestine (in the serosal fluid of the jejunum and ileum), but not elenolic acid [41]. Similarly, only the conjugated forms of M2 were detected in the stomach, intestinal and caecum content, and feces of rats fed for 21 days with a diet supplemented with an extract composed mainly of OLEA [27], although elenolic acid was found in plasma and urine. The absence of elenolic acid in our samples could be explained by its rapid absorption from the hydrolyzed OLEA fraction. In the study of Kano et al. (2016), M2 was also the main metabolite found in the plasma of portal blood after oral administration of OLEA to rats [29]. In addition, M2 was not detected in human urine after EVOO intake [21,22].

Hydrogenated (M3), oxidated (M4), and hydrated (M5) forms of OLEA were detected in lumen, plasma, and ileum tissue (Figure 3). Major phase I enzymes include oxidases (especially monooxygenases), reductases, and hydrolases [10]. The hydrogenation of OLEA (M3) can arise from a reduction reaction catalyzed by NADPH-dependent aldo-keto reductases located in the small intestine epithelium. The reduction of aldehydes to primary alcohols can occur in OLEA because it contains the dialdehydic form of the linked elenolic acid [42]. In addition, the intestinal redox potential provides a reducing environment due to low oxygen tension, whereas oxidation is favored in tissues such as the liver [43]. Pinto et al. [41] proposed a structure for M3 but they were unable to confirm which of the carbonyl functional groups had undergone reduction, as the reaction at either site yielded similar fragmentation patterns. In our study, on the basis of the fragment detected (mass 143), we proposed a hydrogenation of the unsaturated aldehyde, although this cannot be confirmed without an NMR spectrum or the fragmentation analysis of a previously synthesized structure (Figure S3).

Cytochrome P450 enzymes (CYP), monooxygenases found in the epithelium of the small intestine [44], are responsible for the oxidative biotransformation of xenobiotics and other compounds [38,45]. In our work, the OLEA derivatives M4 and M5 could have been produced by the microbiome, as many bacterial CYP are soluble [46], or by the CYP expressed in the enterocytes, as shown in our luminal and tissue samples (Figure 3). However, non-CYP-mediated oxidative reactions can play an important role in the metabolism of xenobiotics [47]. Regarding carboxylic acids, as they are products of aldehyde oxidation, they could also be generated by aldehyde dehydrogenase enzyme catalysis. While only traces of M5 were detected in the TM (stability study), the high amount in both lumen and plasma samples indicates a metabolic reaction during its transport across the intestinal membrane. In 2010, Garcia-Villalba and co-workers found the same OLEA derivatives (M3, M4, and M5) in human urine after olive oil intake [21]. Although these derivatives were not found in plasma and urine samples of healthy volunteers in the study of Silva and co-workers [22], a hydrated OLEA metabolite was observed (OLE + CH_3_ + H_2_O + glucuronide). In fact, the metabolic profile of phenolic compounds from EVOO has been previously studied [23,26,41], but OLEA derivatives have not yet been reported.

### 3.4. Phase II Metabolism

Phase II biotransformation reactions, also known as conjugation reactions, generally serve as a detoxifying step in xenobiotic metabolism, increasing hydrophilicity and therefore excretion, as well as the metabolic inactivation of pharmacologically active compounds [48,49]. Phase II derivatives detected in plasma (M7 and M9), lumen (M7 and M9-M11), and ileum tissue samples (M6, M8, M9, and M11) are presented in Figure 3. The main product of phase II biotransformation reactions in all the samples was M9, with the highest relative abundance in plasma at 45–60 min, and in lumen samples at 55–60 min (*p* < 0.05).

It is well known that the enzyme that catalyzes *O*-methylation is catechol-*O*-methyltransferase, which mediates the transfer of a methyl moiety from the *S*-adenosyl-L-methionine cofactor to a hydroxyl group on the xenobiotic [50]. In rats and humans [51], catechol-*O*-methyltransferase is most active in the liver, kidney, intestine, and brain [52]. In agreement with the computational study carried out by Cuyàs et al. (2019), which concluded that meta-methylation at the O5 position of the catechol residue of OLEA occurs preferentially, we proposed the methylated derivative M6 (Figure 2) [50]. Among the three methylated metabolites, M6 and M8 were found in ileum tissue samples but not in the plasma and lumen (Figure 3), whereas M7 (OLEA + OH + CH_3_) was detected in plasma and, after being secreted by an efflux membrane protein, in lumen samples. Previously reported results for these methylated forms are contradictory, being detected in human urine samples by Garcia-Villalba et al. [21] but not in other studies in rats [26,29,41]. Differences in experimental procedures may explain these discrepancies.

The glucuronidation reaction consists of transferring a glucuronyl moiety from the co-substrate UDP-glucuronic acid to one or more electrophilic groups of a hydrophobic molecules. The family of uridine diphosphate (UDP) glucuronosyltransferases are the enzymes that catalyze this reaction [53]. Glucuronidation often occurs as a secondary step after the production of primary metabolites in phase I reactions such as hydrolysis, hydroxylation, and dealkylation [54], as can be observed in M9 and M10, in which a glucuronic acid is attached to the hydrated and hydrogenated OLEA. Similarly, M11 arises from the addition of a methyl group and a glucuronic acid in a previously hydrated molecule of OLEA. M11 was detected in lumen and ileum tissue samples but not in plasma. The glucuronidation of hydrogenated OLEA has been previously reported in perfused segments of jejunum and ileum in rats [41]. Two different studies in humans obtained similar results: García-Villalba et al. [21] detected all glycoconjugates (M9, M10, and M11), while Silva et al. [22] only found M9 and M11. The bioavailability of phenolic compounds in EVOO depends not only on their concentration but also other dietary components and the individual genomic profile, which can affect enzymatic activity involved in the digestion and metabolism processes [22]. Polymorphism of conjugation enzymes or individual variations in digestive enzymes or bile salts could underlie the variations observed [1,2,17]. The aforementioned studies on humans reported three additional glycoconjugates (OLEA + glucuronide, OLEA + CH_3_ + glucuronide, and OLEA + CH_3_ + OH + glucuronide) not identified in our work, which may have been due to differences in the species and model used to evaluate the intestinal metabolism (in vivo vs. in situ models) [1]. The presence of these metabolites in humans can also be explained by the hepatic metabolism that OLEA or its derivatives may undergo after absorption.

**Figure 4:**
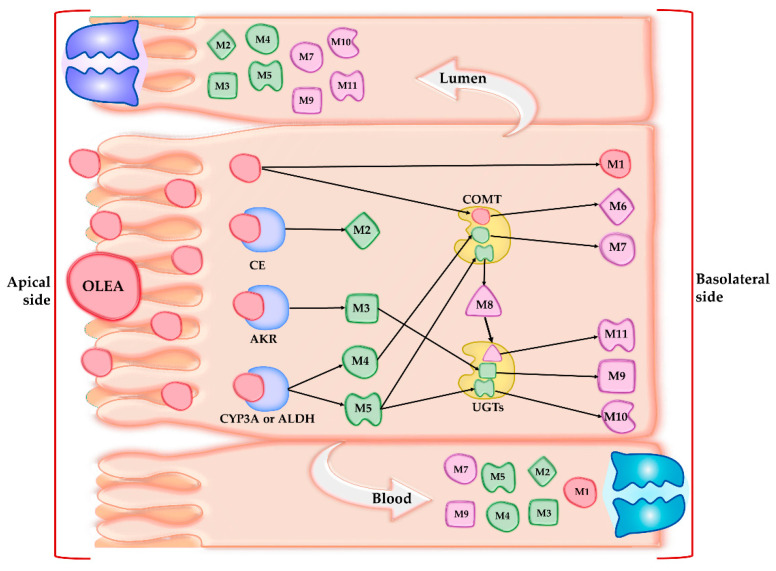
Tentative interactions of oleacein (OLEA) with metabolic enzymes and transporters. CE: carboxylesterases; AKR: aldo-keto reductases; CYP3A: subfamily of cytochrome P450 enzymes; UGTs: glucuronosyltransferases; COMT: catechol-*O*-methyltransferase; ALDH: aldehyde dehydrogenase.

In the present work, the observed phase I (M2-M5) and phase II (M7 and M9) OLEA derivatives in both lumen and plasma samples can be attributed to the presence of specific membrane transporters expressed in the apical (MDR1, BCRP, MRP2) or basal membrane (MRP1) of the enterocytes [55]. The OLEA derivatives recognized by these efflux transporters would thus be secreted to the intestinal lumen. On the basis of the results obtained, Figure 4 depicts the metabolic fate of OLEA in the small intestine, showing possible interactions with metabolic enzymes and carriers during transport across the enterocyte.

### 3.5. Absorption Study

To investigate the intestinal permeability of OLEA, we carried out a comparative study with the anti-inflammatory drug naproxen. A highly permeable standard [55], naproxen has an oral bioavailability close to 100% [33] and was tested with the same in situ perfusion technique and conditions as OLEA.

The SPIP model is used in general screening for the intestinal membrane permeability of orally administered drugs and xenobiotics and to predict the effective permeability coefficient (*P*_eff_). In this study, the ileum permeability of the tested compounds was based on luminal disappearance as well as appearance in mesenteric blood plasma, a suitable option for substances with low membrane permeability, as the differences in perfusate concentrations may be too small to determine accurately [8]. To obtain information about the intestinal metabolism of OLEA (see Section 3.3 and Section 3.4), we analyzed perfusate and plasma samples for potential derivatives. The results of the absorption study are presented in Table 2, together with previously reported data for comparison. As can be observed, the *P*_eff_ values we obtained for naproxen agreed with those in the literature, indicating the validity of the methodology employed. However, as the variability between laboratories is relatively high, the data from individual studies should be interpreted separately [56].

Despite the broad range of promising biological effects of secoiridoids from EVOO [17], the *P*_eff_ value of OLEA has not been previously reported. The mean permeability ratio (*P*_eff_) of OLEA/naproxen was 1.24, without significant differences between them (*p* > 0.05), which indicates that the intestinal permeability of OLEA is comparable with that of the highly permeable standard. No significant differences were found for the percentage of drug absorbed (48.98 ± 12.27% and 43.96 ± 7.58% for OLEA and naproxen, respectively). These results are coherent with the lipophilicity (expressed as log P (octanol/water)) and molecular weight of OLEA (1.53 [60]; 1.02 [16], and 320.3 g/mol, respectively), which favor intestinal membrane transport by passive mechanisms [61].

**Figure 5:**
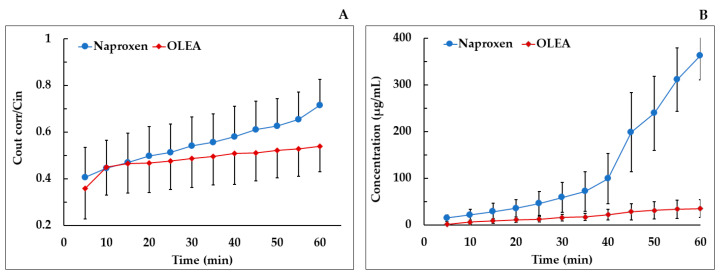
(**A**) Plot of the mean concentration ratio of the corrected outlet and inlet concentrations vs. time for naproxen and OLEA in SPIP in rats; (**B**) mean mesenteric plasma concentration of naproxen and OLEA vs. time. Error bars represent S.D.

In the mesenteric blood plasma, in our experimental conditions, the *P*_app_ of naproxen was significantly lower than that of OLEA (Figure 5B). As naproxen has a complete oral bioavailability, this result could be explained by a higher retention in or interaction with the lipid membranes (apical and basolateral), attributable to its high lipophilicity (log P = 3.18 [62]) and molecular structure, rather than a presystemic intestinal metabolism. Other authors have described an affinity of naproxen and other nonsteroidal anti-inflammatory drugs (NSAIDs) for the phosphatidylcholine of biological membranes [63,64]. It would therefore have been interesting to extend the study time from 60 min to, for example, 90 min.

To the best of our knowledge, this is the first study to report the intestinal and apparent permeability coefficients of OLEA in rats. Pinto et al. (2011), who investigated the metabolism of OLEA in an in vitro intestinal preparation from rats, also described its transport through the small intestine, observing OLEA in the receptor medium in an in vitro assay with Caco-2 cells [41]. Kano et al. (2016), who studied the absorption, metabolism, and excretion of OLEA after oral administration (300 mg/kg) in rats, did not observe OLEA in the portal plasma, possibly because its detection was hindered by binding to plasma components such as serum albumin, serum lipoprotein, and glycoprotein [29]; phenolic compounds and their metabolites are known to form complexes with plasma proteins [65]. Although further investigation is required, the amount of OLEA metabolites found in the perfusate and plasma in our study suggests that the bioavailability of this phenolic compound is incomplete. In conclusion, the important role of the small intestine in the bioavailability of OLEA has been demonstrated in terms of absorption and membrane transport as well as metabolic reactions that contribute to its elimination.

## 4. Conclusions

This is the first in situ study to simultaneously assess the absorption and intestinal metabolism of OLEA in rats. The SPIP model was used to determine the intestinal effective permeability of OLEA on the basis of its disappearance from the intestinal segment and its appearance in mesenteric blood. The range and abundance of metabolites found in the perfusate and plasma suggest that the oral bioavailability of OLEA in rats is incomplete. The results indicate that the small intestine plays an important role in the bioavailability of OLEA, considering its high intestinal permeability and the metabolic reactions that contribute to its elimination. The metabolites arising from hydrolysis (M2) and hydroxylation (M4) were the main circulating metabolites of OLEA detected in plasma and the lumen. The higher metabolite levels in plasma suggests that the intestinal metabolism of OLEA occurs mainly during the transport of the compound across the intestinal membrane.

## Figures and Tables

**Table 1:** Identification of OLEA and its metabolites in lumen, plasma, and ileum tissue samples by LTQ–Orbitrap–MS.

	Compound	Precursor Ion Measured *m*/*z* [M − H]^−^	Tentative Formula [M − H]^−^	Mass Error (ppm)	RT (min)	MS/MS
M1	OLEA	319.1184	C_17_H_19_O_6_	0.785	6.81	153/183
Phase	I					
M2	OH-TY	153.0554	C_8_H_9_O_3_	0.779	3.67	123
M3	OLEA + H_2_	321.1337	C_17_H_21_O_6_	0.435	7.05	185/199/143
M4	OLEA + OH	335.1128	C_17_H_19_O_7_	0.271	6.82	131/199
M5	OLEA + H_2_O	337.1282	C_17_H_21_O_7_	0.021	6.69	201/133
Phase	II					
M6	OLEA + CH_3_	333.1348	C_18_H_21_O_6_	0.835	8.51	167
M7	OLEA + OH + CH_3_	349.1277	C_18_H_21_O_7_	−0.479	7.38	167/199
M8	OLEA + H_2_O + CH_3_	351.1445	C_18_H_23_O_7_	1.771	7.20	215/167
M9	OLEA + H_2_ + Glucu	497.1665	C_23_H_29_O_12_	1.247	6.53	199/329
M10	OLEA + H_2_O + Glucu	513.1621	C_23_H_29_O_13_	1.833	6.43	329/215
M11	OLEA + H_2_O + CH_3_ + Glucu	527.1743	C_24_H_31_O_13_	0.963	6.50	343/201

RT: retention time; Glucu: glucuronic acid; OH-TY: hydroxytyrosol.

**Table 2:** Intestinal effective permeability coefficient (*P*_eff_), apparent permeability coefficient (*P*_app_), and percentage of absorption after SPIP (mean ± SD, *n* = 4) for OLEA and the reference standard naproxen. Reported data are also included.

Compound	Segment	*P*_eff_ × 10^−4^ (cm/s) ± SD	*P*_app_ × 10^−4^ (cm/s) ± SD	Absorption (%)	Study
OLEA	Ileum	1.83 ± 0.18	0.607 * ± 0.202	48.98 ± 12.27	Current study
Naproxen	Ileum	1.47 ± 0.44	0.19 ± 0.018	43.96 ± 7.58	Current study
		1.17 ± 0.23			[57]
		1.78 ± 0.52			[56]
	Jejunum	1.17 ± 0.23			[58]
		1.19 ± 0.12			[33]
		1.47 ± 0.25			[56]
		2.10 ± 0.41			[59]
	Colon	2.06 ± 1.04			[56]

* *p* < 0.05 differences OLEA vs. naproxen (Mann–Whitney *U* test).

## Data Availability

Data is contained within the article or supplementary material. The data presented in this study are available in Figure 1, Figure 2, Figure 3, Figure 4, and Figure 5. In addition, the information is also presented in Table 1 and Table 2.

## Supplementary Materials

The following are available online at https://www.mdpi.com/article/10.3390/pharmaceutics13050719/s1, Figure S1. Concentration of NAP (right *Y*-axis scale) and OLEA (left *Y*-axis scale) in the TM and 37 °C as a function of time in the stability study. Figure S2. Chromatogram and retention time of OLEA and its metabolites. Figure S3. Molecular structure of OLEA and derivatives and proposed fragments with their masses through LQT–Orbitrap–MS analysis.
