# Supplementary material for: Oleacein Intestinal Permeation and Metabolism in Rats Using an In Situ Perfusion Technique
Source: Pharmaceutics. 2021 May 14;13(5):719. doi: 10.3390/pharmaceutics13050719 (PMC8153610; doi:10.3390/pharmaceutics13050719)
Supplement: Supplementary file 1 [file pharmaceutics-13-00719-s001.zip › pharmaceutics-1219271-supplementary.pdf]

pharmaceutics

MDPI

Supplementary Materials: Oleacein Intestinal Permeation and Metabolism in Rats Using an In Situ Perfusion Technique

Lastname Anallely López-Yerena, Maria Pérez, Anna Vallverdú-Queralt, Eleftherios Miliarakis, Rosa M. Lamuela-Raventós and Elvira Escribano-Ferrer

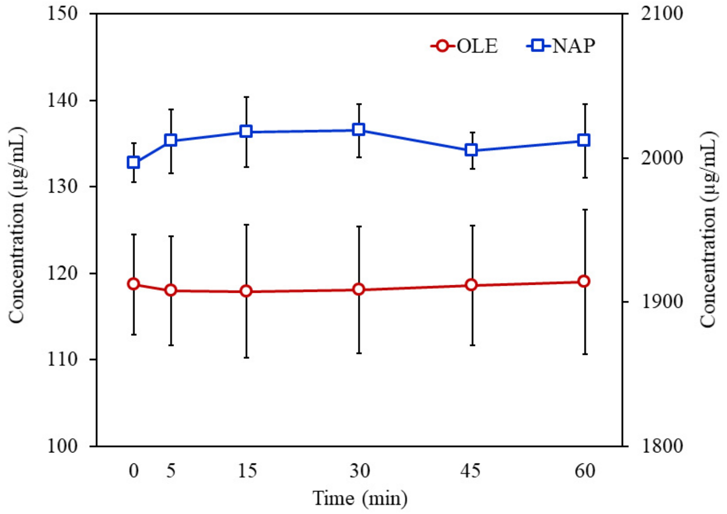

Figure S1. Concentration of NAP (right Y-axis scale) and OLEA (left Y-axis scale) in the TM and 37°C as a function of time in the stability study.

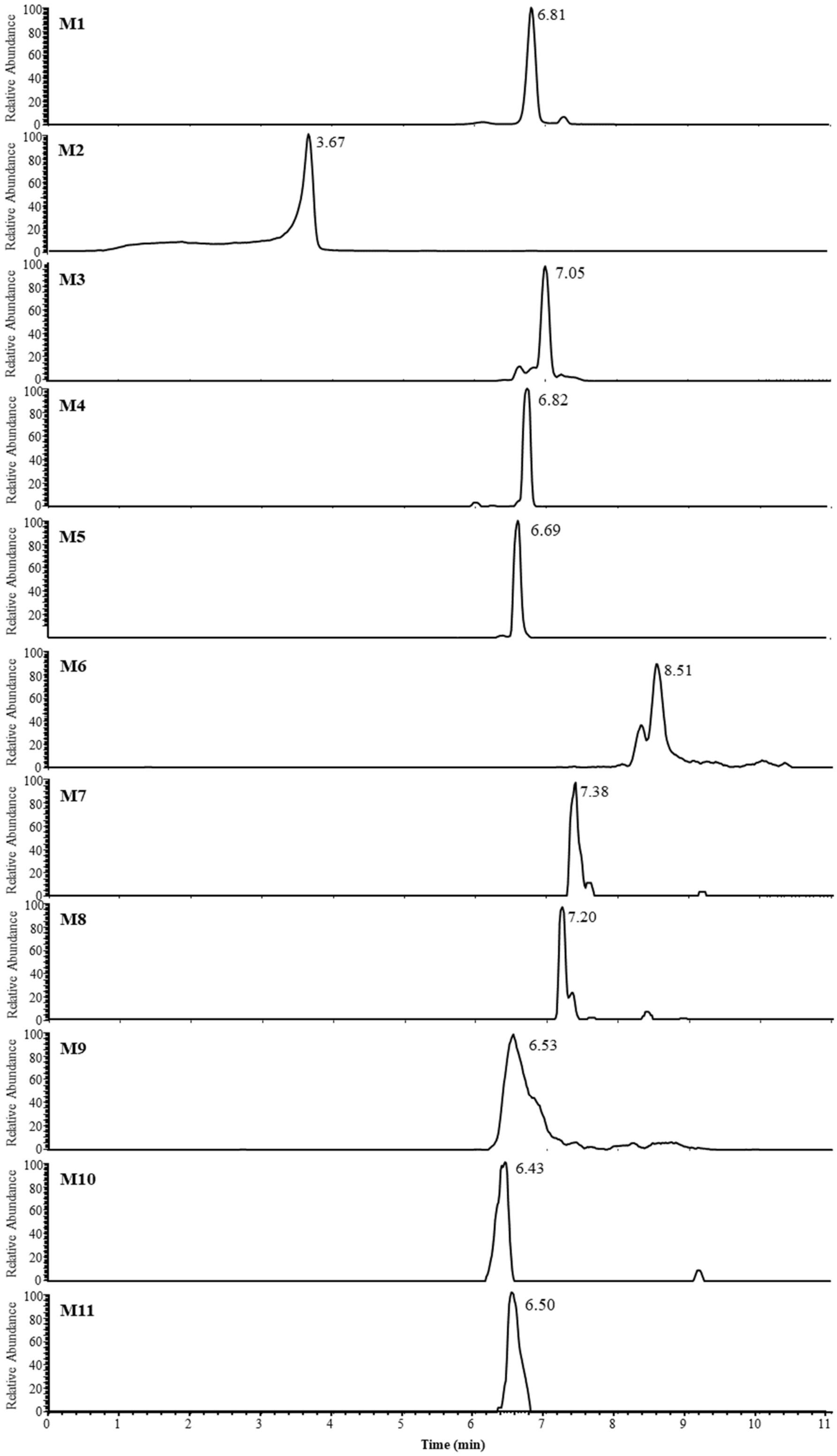

Figure S2. Chromatogram and retention time of OLEA and its metabolites.

M1

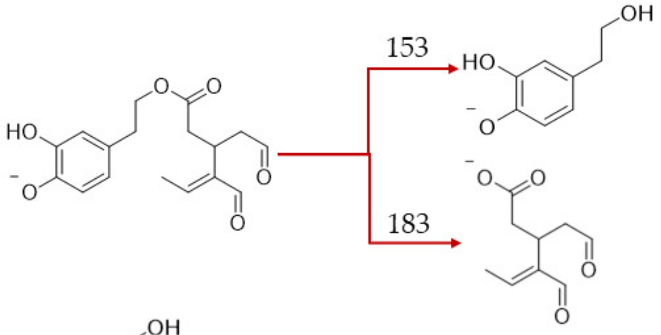

M2

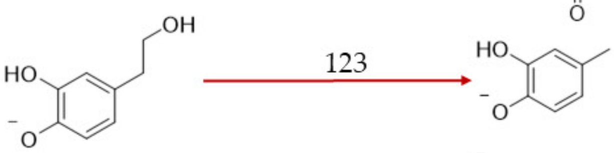

M3

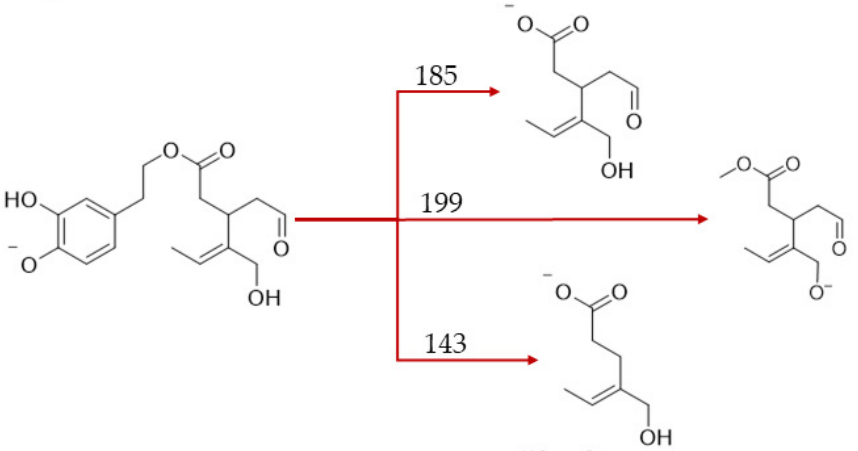

M4

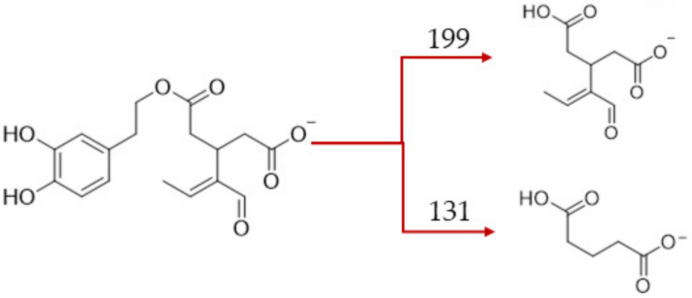

M5

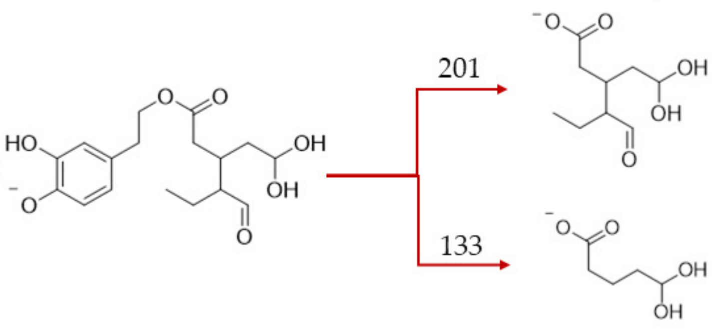

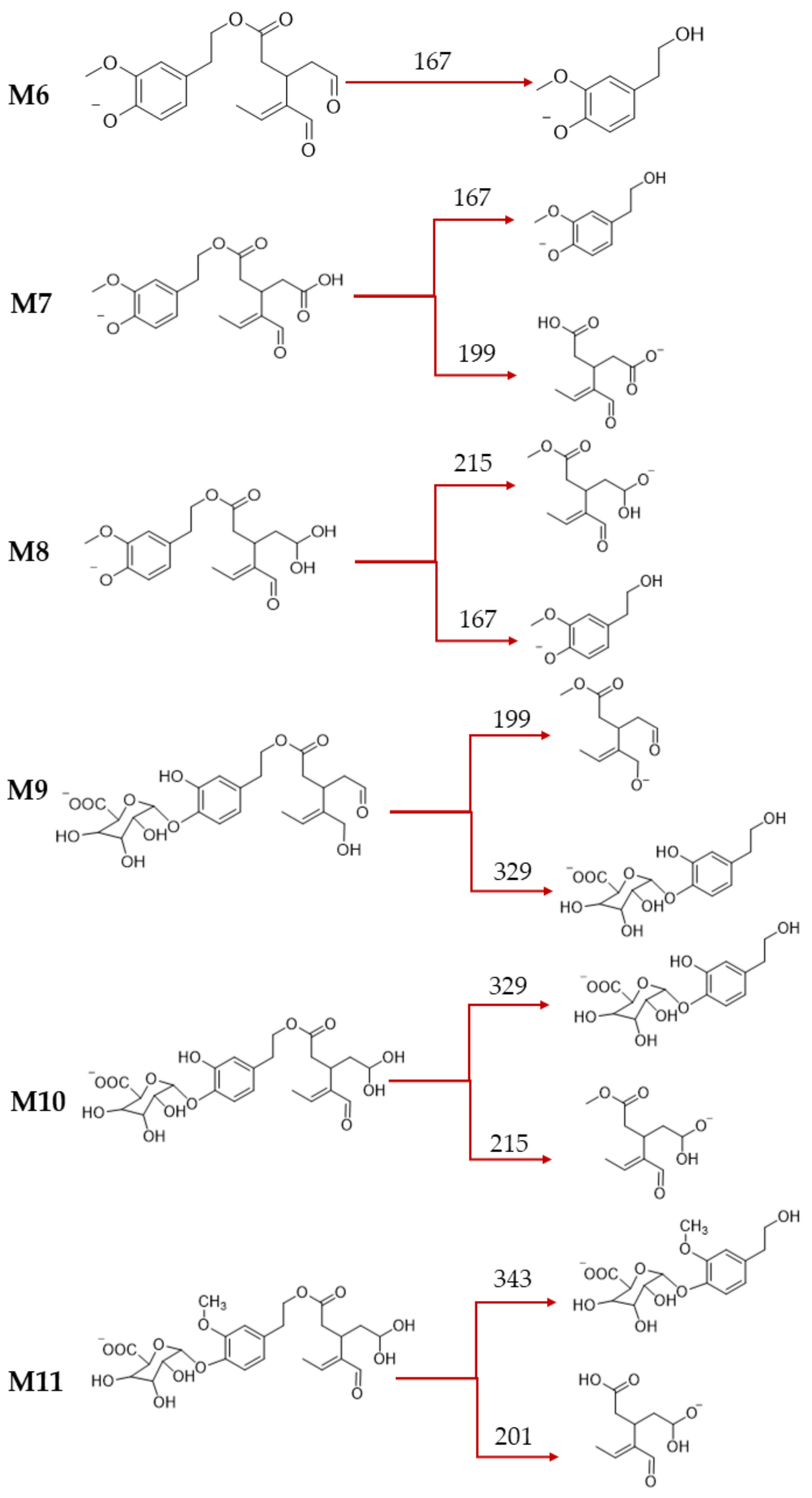

Figure S3. Molecular structure of OLEA and derivatives and proposed fragments with their masses through LQT-Orbitrap-MS analysis.
